# Time-dependent effects of cortisol on selective attention and emotional interference: a functional MRI study

**DOI:** 10.3389/fnint.2012.00066

**Published:** 2012-08-28

**Authors:** Marloes J. A. G. Henckens, Guido A. van Wingen, Marian Joëls, Guillén Fernández

**Affiliations:** ^1^Donders Institute for Brain, Cognition and Behaviour, Radboud University NijmegenNijmegen, Netherlands; ^2^Department of Neuroscience and Pharmacology, Rudolf Magnus Institute, University Medical Center UtrechtUtrecht, Netherlands; ^3^Department of Psychiatry, Academic Medical Center, University of AmsterdamAmsterdam, Netherlands; ^4^Department of Cognitive Neuroscience, Radboud University Nijmegen Medical CentreNijmegen, Netherlands

**Keywords:** corticosteroids, emotional interference, attention, functional MRI, amygdala, prefrontal cortex, cuneus, insula

## Abstract

Acute stress is known to induce a state of hypervigilance, allowing optimal detection of threats. Although one may benefit from sensitive sensory processing, it comes at the cost of unselective attention and increased distraction by irrelevant information. Corticosteroids, released in response to stress, have been shown to profoundly influence brain function in a time-dependent manner, causing rapid non-genomic and slow genomic effects. Here, we investigated how these time-dependent effects influence the neural mechanisms underlying selective attention and the inhibition of emotional distracters in humans. Implementing a randomized, double-blind, placebo-controlled design, 65 young healthy men received 10 mg hydrocortisone either 60 min (rapid effects) or 270 min (slow effects), or placebo prior to an emotional distraction task, consisting of color-naming of either neutral or aversive words. Overall, participants responded slower to aversive compared to neutral words, indicating emotional interference with selective attention. Importantly, the rapid effects of corticosteroids increased emotional interference, which was associated with reduced amygdala inhibition to aversive words. Moreover, they induced enhanced amygdala connectivity with frontoparietal brain regions, which may reflect increased influence of the amygdala on an executive network. The slow effects of corticosteroids acted on the neural correlates of *sustained* attention. They decreased overall activity in the cuneus, possibly indicating reduced bottom-up attentional processing, and disrupted amygdala connectivity to the insula, potentially reducing emotional interference. Altogether, these data suggest a time-specific corticosteroid modulation of attentive processing. Whereas high circulating corticosteroid levels acutely increase emotional interference, possibly facilitating the detection of threats, a history of elevation might promote sustained attention and thereby contribute to stress-recovery of cognitive function.

## Introduction

Stress has profound influence on the brain's attentional resources. When exposed to an acutely stressful situation, the brain shifts into a mode of hypervigilant processing in which the detection and assessment of potential threats is optimized by prioritized sensory processing (de Kloet et al., 2005; van Marle et al., 2009), and the amygdala, key modulator of vigilance and emotional processing in the brain (Phelps and Ledoux, 2005), is activated (van Marle et al., 2009). This surge in vigilance in immediate response to stress is thought to be mediated by the central release of norepinephrine (NE) by tonic activation of the locus coeruleus (LC) (Aston-Jones and Cohen, 2005; Valentino and Van Bockstaele, 2008; Cousijn et al., 2010). This state of hypervigilance is highly adaptive and enhances chances of survival during stressful situations, but it comes at the cost of specificity (van Marle et al., 2009), impaired selective attention (Tanji and Hoshi, 2008; Henderson et al., 2012) and increased susceptibility to distraction (Skosnik et al., 2000; Braunstein-Bercovitz et al., 2001; Aston-Jones and Cohen, 2005), resulting from impaired prefrontal cortex (PFC) processing underlying executive functioning (Arnsten, 2009; Qin et al., 2009) and exhaustion of attentional resources (Sato et al., 2012). It might cumulate in stress-related disorders such as depression and post-traumatic stress disorder (PTSD), which are characterized by an attentional bias toward negative emotional information (Williams et al., 1996). Therefore, normalization of attentional processing some time after the stressful event is very important for well-being. Notably, these disorders are characterized by aberrant corticosteroid signaling (Yehuda et al., 2001).

Corticosteroids, released in response to stress as the end-product of the hypothalamic-pituitary-adrenal (HPA) axis, are well-known modulators of human cognition. The hormones exert their actions upon binding of the mineralocorticoid (MR) and glucocorticoid receptor (GR), which are abundantly expressed in the brain (Sapolsky et al., 1983; Reul and de Kloet, 1985; de Kloet, 1991). Recent research in rodents has indicated that corticosteroid-binding can induce both rapid non-genomic and slow genomic effects by acting on receptors that are respectively located in the plasma membrane and in the nucleus (Di et al., 2003; Karst et al., 2005; Wiegert et al., 2005). These distinct temporal pathways are thought to serve different functions (Joels et al., 2006, 2011). The rapid actions of corticosteroids on the one hand, have been suggested to work in concert with (and amplify) the effects of catecholamines (Roozendaal et al., 2006; Joels and Baram, 2009) to optimize rapid adaptive behavior by relocating neural resources away from higher-order cognitive processing regions in the PFC to the limbic structures (Diamond et al., 2007). Therefore, they might boost the effects of catecholamines on attentional processing, increasing emotional interference. The slow corticosteroid-induced genomic cascade is on the other hand thought to be responsible for the regulation of the stress response and the restoration of homeostasis in the aftermath of stress (de Kloet et al., 2005; Henckens et al., 2010, 2011c).Thereby, the slow corticosteroid effects might contribute to the normalization of attentional processing in the aftermath of stress. However, these time-dependent effects of corticosteroids on the neural substrates of selective attention have never been tested.

Here, we set out to investigate the time-dependent effects of corticosteroids on the neural correlates of selective attentional processing. In a randomized, double-blind, placebo-controlled design, 65 young healthy men received 10 mg hydrocortisone either 60 min (to target the rapid corticosteroid effects) or 270 min (slow corticosteroid effects), or placebo prior to functional MRI scanning. Selective attention was assessed by means of an emotional distraction task, in which participants were asked to identify the font color of neutral and highly aversive words as fast and accurate as they could (Mathews and MacLeod, 1985; McKenna, 1986). Proper selective attention is critical for task-execution, since it requires participants to focus on just one source of information for processing (i.e., font color) while ignoring competing information, including word meaning (e.g., emotion). It is well-known that under such competitive conditions, the presence of emotionally salient information disrupts the ability to attend selectively to the task-relevant information (Arnsten and Goldman-Rakic, 1998; Dolcos and McCarthy, 2006; Dolcos et al., 2011). Typically, this results in slower reaction times and lower accuracy for color naming of emotional words relative to neutral words, which serves as a measure of emotional interference. By measuring the corticosteroid effect on emotional interference induced by the emotional, attention-grabbing distracters (Bishop, 2008; Wingenfeld et al., 2009), this task enabled us to assess corticosteroid effects on selective attention. Moreover, this task enabled us to assess corticosteroid effects on sustained attention, i.e., one's ability to maintain a consistent response during continuous (i.e., repetitive) task performance. In other words, it measures the ability to keep the selective attention maintained over time (McDowd, 2007). Since sustained attention is required to complete any cognitively planned activity, here task execution, it could be assessed by analyzing overall task performance, regardless of the emotional valence of the words.

## Materials and methods

### Participants

Seventy-two young (age range 18–29, median 21), right-handed, Dutch speaking, healthy male volunteers gave written informed consent to participate in the study. Women were excluded from participation, since previous research has indicated that women respond differently to hydrocortisone than men, both in behavior (Andreano and Cahill, 2006; Bohnke et al., 2010) and brain activation (Stark et al., 2006; Merz et al., 2010). Moreover, their response to hydrocortisone is modulated by oral contraceptive use and varies over the menstrual cycle (Merz et al., 2011). Therefore, in order to reduce variance we here recruited the group with the most stable response to hydrocortisone. Furthermore, individuals who met any of the following criteria were excluded from participation: history of head injury, autonomic failure, history of or current psychiatric, neurological, or endocrine disorders, current periodontitis, acute inflammatory disease, acute peptic or duodenal ulcers, regular use of corticosteroids, treatment with psychotropic medications, narcotics, beta-blockers, steroids, or any other medication that affects central nervous system or endocrine systems, medical illness within the three weeks prior to testing, self reported mental or substance use disorder, daily tobacco or alcohol use (or experienced inconvenience in refraining from these activities for three days), exercising at the professional level, regular night shift work, or current stressful episode or major life event. Four participants were excluded from analyses because of unreliable cortisol manipulation [abnormal basal cortisol levels (1 × placebo) or no elevation in salivary cortisol level in response to CORT intake (2 × rapid CORT, 1 × slow CORT)], and another three participants because of insufficient task performance (based on outlier analyses (>3 SD below average performance; 2 × placebo, 1 × slow CORT). Thus, the results comprise data of 21 men in the placebo group, and 22 men in the rapid CORT and 22 men in the slow CORT group. The study was approved by the local ethics committee (CMO region Arnhem-Nijmegen, Netherlands) and executed in accordance with the declaration of Helsinki.

### Study design

#### Prior to arrival

To minimize differences in baseline cortisol levels we instructed participants not to use any recreational drugs for three days and to refrain from drinking alcohol, exercising, and smoking for 24 h prior to the appointment. Furthermore, participants were requested not to brush their teeth, floss, or eat and drink anything but water for 1 h prior to the session enabling adequate saliva sampling for cortisol assessment. They were asked to take a light lunch and do so no later than 1 h before arrival; their lunch could not contain any citrus products, coffee, tea, milk, or sweets (Maheu et al., 2005). Throughout the entire study period, participants were only given water to drink, except for a scheduled lunch at *t* = −180 min.

#### Arrival

To reduce the impact of diurnal variation in cortisol levels, all testing was performed in the afternoon, between 12 pm (±30 min) and 6:00 pm (±30 min), when hormone levels are relatively stable. Upon arrival participants received an information brochure about the procedure, they gave informed consent, and completed an intake questionnaire to ensure that in- and exclusion criteria were met. Thirty minutes after arrival, a first saliva sample was taken, followed by another one 15 min later, in order to measure a reliable baseline level. Participants were asked to complete a first Profile of Mood States (POMS) questionnaire (Reddon et al., 1985; Wald and Mellenbergh, 1990; de Groot, 1992), after which they briefly trained the emotional distraction task to ensure proper performance during scanning. Immediately after the second saliva sample (at *t* = −270 min) participants received the first capsule. During the entire period (~4 h) prior to scanning, participants waited in a quiet room where they were free to conduct any activities except for anything potentially arousing (e.g., video games). At 60 min prior to the emotional distraction task participants were asked to complete another POMS questionnaire, and received the second capsule. Both drug capsules, containing either 10 mg CORT or placebo (cellulose), were administered orally. This dose is known to elevate salivary cortisol levels to moderate to high stress levels (Kirschbaum et al., 1996; Morgan et al., 2000; Tops et al., 2003), and has been shown to be successful in the induction of corticosteroid effects on declarative memory (Kirschbaum et al., 1996; Tops et al., 2003). Depending on the group to which the participant was (randomly) assigned he received either; the 1st capsule containing placebo, the 2nd containing placebo (placebo group); the 1st capsule CORT, the 2nd placebo (slow CORT group); or the 1st capsule placebo, the 2nd CORT (rapid CORT group). The experiment described here was part of a larger study into the time-dependent effects of corticosteroids on emotional and cognitive brain function. Results on the other tasks have been reported elsewhere (Henckens et al., 2010, 2011a,b,c).

#### Emotional interference task

**Figure 1:**
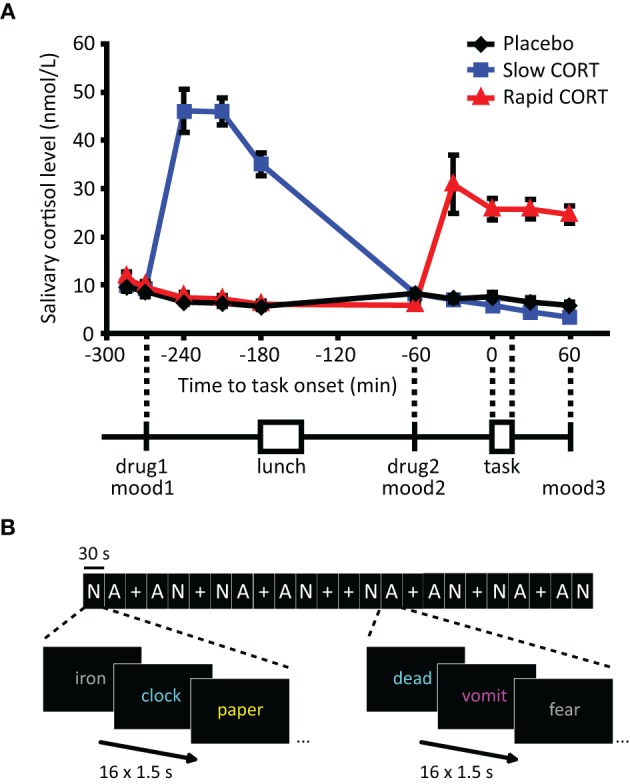
**Salivary cortisol data and experimental design. (A)** Participants received two capsules (drug1 and drug2) containing either 10 mg of hydrocortisone (CORT) or placebo at different time points before the emotional distraction task. Hydrocortisone intake significantly elevated salivary cortisol levels in both hydrocortisone administration groups to levels observed during moderate-to-severe stress (Morgan et al., 2000). **(B)** The emotional distraction task consisted of 30 s-blocks of neutral (N) or aversive (A) words or fixation (+). Participants were requested to button press as fast as possible for the color in which the presented words were displayed. Mood: POMS questionnaire (Reddon et al., 1985; Wald and Mellenbergh, 1990; de Groot, 1992). Error bars represent S.E.M. N.B. In reality Dutch words were used, the words in Figure 1B only serve an illustrative purpose.

The emotional interference task started 60 min after administration of the second capsule (at *t* = 0 min) (Figure 1A). In brief, series of colored words were presented to the participants, and they were asked to press one of four buttons as fast as possible for the color in which the word was displayed. Words were presented either in blue, magenta, yellow, or gray, which was counterbalanced across subjects, and colors were matched in luminosity. Colors were chosen for their distinctiveness, while any associations with go- or stop-signals (i.e., green and red) were excluded to prevent their confounding effects on reaction times, inducing increased variability between colors. Participants used both their index- and middle fingers to respond, ensuring proper fast responding.

Words belonged to one of two categories, neutral or aversive, and were selected for the emotional valence and arousal ratings of their translation in English in the Affective Norms for English Words (ANEW) database (Bradley and Lang, 1999). Aversive words were selected for their high arousal and low valence, as rated on a 1–9 scale using the Self-Assessment Manikin (SAM) scales (Bradley and Lang, 1994), while neutral words were selected for their low arousal and neutral valence ratings. Subsequently, words were translated in Dutch and categories were matched on average word length [mean ± SEM; 6.63 ± 1.62 (neutral), 6.84 ± 1.85 (aversive)] and word form frequency [1006.56 ± 103.77 (neutral), 920.60 ± 88.97 (aversive)], and lemma frequency [1528.63 ± 154.16 (neutral), 1307.12 ± 135.11 (aversive)] based on the Dutch lexical database CELEX (Baayen et al., 1995). In total, 128 words of each category were selected. To confirm proper valence and arousal levels of these Dutch words, all participants were asked to rate the words one day after the experiment, using the SAM scales (Bradley and Lang, 1994). These ratings confirmed word categorization. The sets of aversive and neutral words differed on arousal [mean ± SEM; 3.79 ± 0.07 (aversive), 1.70 ± 0.04 (neutral), *t*_(254)_ = 24.29, *p* < 0.001] and valence [3.13 ± 0.06 (aversive), (5.31 ± 0.04 neutral), *t*_(254)_ = −32.37, *p* < 0.001].

The total task lasted 12 min and consisted of eight blocks of each category (containing 16 words presented for 1.5 s, 0.15 s ISI, 3.6 s inter-block fixation), supplemented with eight fixation blocks. Words were presented in a pseudo-random color (immediate color repetition was not allowed). Blocks were presented in a mirrored design avoiding covariation with linear drift, and adjacent blocks of the same emotion were avoided (Figure 1B). To ensure proper understanding and sufficient performance, participants had twice a short two-block practice of nonsense words (random letters); once earlier that day outside the MRI scanner (at *t* = −270 min), and once inside the scanner immediately prior to the actual task (*t* = 0 min). Since participants were instructed to respond as fast and as accurately as possible, task performance was assessed both in terms of reaction times and error rates (Swick and Jovanovic, 2002; Wagner et al., 2006; Weiss et al., 2007; Kertzman et al., 2010). Sustained attentional performance was defined by overall performance on the task combining both neutral and aversive trials, whereas selective attention (i.e., emotional interference) was assessed by contrasting performance between these trials (aversive vs. neutral). The session ended with a high resolution anatomical scan.

### Physiological and psychological measures

#### Saliva collection and analysis

Cortisol levels were measured from saliva at ten time points: two baseline measurements at the beginning of the experimental day (*t* = −285, –270 min), and eight samples thereafter (*t* = −240, −210, −180, −60, −30, 0, 30, and 60 min) to assess cortisol changes throughout the experiment. Saliva was collected using a commercially available collection device (Salivette®, Sarstedt, Germany). For each sample, the participant first placed the cotton swab provided in each Salivette tube in his mouth and chewed gently on it for 1 min to produce saliva. The swab was then placed back in the Salivette tube, and the samples were stored in a freezer at −25°C until assayed. Laboratory analyses were performed at the Department of Biopsychology, TU Dresden, Germany. After thawing, Salivettes were centrifuged at 3000 rpm for 5 min, which resulted in a clear supernatant of low viscosity. Salivary free cortisol concentrations were subsequently measured using a commercially available chemiluminescence-immuno-assay (CLIA) with high sensitivity of 0.16 ng/ml (IBL, Hamburg, Germany).

#### Mood state

To determine whether hydrocortisone administration led to psychological side-effects, mood state was assessed using the POMS questionnaire (Reddon et al., 1985; Wald and Mellenbergh, 1990; de Groot, 1992) at three time points: at the beginning of the experiment (*t* = −285 min), just prior to the intake of the second capsule (*t* = −60 min), and at the end of the experiment (*t* = 60 min).

### Physiological and psychological statistical analysis

Behavioral and physiological data were analyzed in SPSS 15.0 (SPSS, Inc., Chicago, IL, USA) using repeated measured ANOVAs with drug condition (placebo vs. rapid CORT vs. slow CORT) as between subject factor. Due to the high levels of skewness and kurtosis of the POMS questionnaire (Reddon et al., 1985; Wald and Mellenbergh, 1990; de Groot, 1992), mood data were analyzed using non-parametric tests. Changes over time in mood state were assessed by Friedman tests, and Mann–Whitney U tests were used to assess potential drug effects on mood. Alpha was set at 0.05 throughout.

### MRI acquisition

At approximately 4.5 h after arrival, participants were taken to the scanner room and the procedures were explained. Participants lay supine in the scanner and viewed the screen through a mirror positioned on the head coil. They were asked to lie as still as possible, keep their eyes open, and look directly and continuously at the center of the screen in front of them.

Participants were scanned by a Siemens (Erlangen, Germany) MAGNETOM Avanto 1.5 Tesla MRI scanner equipped with an 8-channel head coil. A series of blood oxygenation level dependent (BOLD) T2^*^-weighted gradient echo EPI images was acquired with the following parameters: TR = 2340 ms, TE = 35 ms, FA = 90°, 32 axial slices approximately aligned with AC-PC plane, slice matrix size = 64 × 64, slice thickness = 3.5 mm, slice gap = 0.35 mm, FOV = 212 × 212 mm^2^. Owing to its relatively short TE, this sequence yields optimal contrast-to-noise ratio in the medial temporal lobes. High resolution anatomical images were acquired for individuals by a T1-weighted 3D Magnetization-Prepared RApid Gradient Echo (MP-RAGE) sequence, which employed the following parameters: TR = 2250 ms, TE = 2.95 ms, FA = 15°, orientation: sagittal, FOV = 256 × 256 mm^2^, voxel size = 1.0 mm isotropic.

### fMRI data analysis

Data were analyzed using Statistical Parametric Mapping software (SPM5; UCL). The first five EPI volumes were discarded to allow for T1 equilibration. Before analysis, the images were motion corrected using rigid body transformations and least sum of squares minimization. Subsequently, they were temporally adjusted to account for differences in sampling times across different slices. All functional images were then coregistered with the high-resolution T1-weighted structural image using normalized mutual information maximization. The anatomical image was subsequently used to normalize all scans into Montreal Neurological Institute (MNI) 152 space. All functional images were resampled to a voxel size of 2 mm isotropic. Finally, all images were smoothed with an isotropic 8 mm full-width-at-half-maximum Gaussian kernel to accommodate residual functional/anatomical variance between subjects. Data were analyzed using a general linear model, in which blocks were modeled based on emotion type. Regressors were temporally convolved with the canonical hemodynamic response function of SPM5. The six covariates corresponding to the movement parameters obtained from the realignment procedure were also included in the model. To reduce unspecific differences between scan sessions, and to correct for any unspecific, global effects of drug intake on hemodynamic response instead of neuronal activation (Desjardins et al., 2001; Peeters and Van Der Linden, 2002), global normalization using proportional scaling was applied. The single subject parameter estimates from each session and condition obtained from the first-level analysis were included in subsequent random-effects analyses. For the second-level analysis, a factorial ANOVA was used, with emotion (neutral vs. aversive) as the within-subject factor, and drug condition (placebo vs. rapid CORT vs. slow CORT) as the between-subject factor.

Statistical tests were family-wise error (FWE) rate corrected (*p* < 0.05) for multiple comparisons at the voxel level for the main effects, and on the cluster-level using a height threshold of *p* < 0.01 for the drug × emotion interaction, depending on the robustness of the effects. Correction for multiple comparisons was done across the entire brain or for regions of interest (ROI) using a small volume correction. Given the abundance of GRs and MRs in the amygdala (de Kloet, 1991) and its involvement in emotional processing (Phan et al., 2002; Ochsner and Gross, 2005), this region was considered ROI. Data concerning the amygdala was corrected for a reduced search volume, defined as a sphere with 4 mm radius, centered on the locus of previously observed stress effects on amygdala responsivity (Ossewaarde et al., 2010).

### Functional connectivity analysis

For connectivity analyses, the time-course of amygdala activity was obtained by extracting the first eigenvariate of the anatomically defined bilateral amygdala [WFU PickAtlas Tool (version 2.4)]. To obtain time-course correlation images irrespective of the experimental conditions, a new statistical model was constructed with the time-course of the amygdala as covariate of interest and the convolved regressors for the experimental conditions and realignment parameters as covariates of no interest, as well as a constant. Time course correlation images were obtained for the amygdala and entered into subsequent random-effects analyses, using a factorial ANOVA with drug condition (placebo vs. rapid CORT vs. slow CORT) as the between-subject factor. Similar to the conventional fMRI analyses, statistical tests were FWE rate corrected (*p* < 0.05) for multiple comparisons at the voxel level for the main effects of amygdala coupling across drug conditions, and on the cluster-level using a height threshold of *p* < 0.01 to assess cortisol effects. Visualizations of activations were created in SPM5 by superimposing statistical parametric maps thresholded at *p* < 0.01 uncorrected (unless specified otherwise) onto a canonical T1-weighted image in a standard MNI 152 space.

## Results

### Physiological and psychological measures

As expected, oral administration of 10 mg hydrocortisone increased salivary cortisol levels to those observed during moderate-to-severe stress (Morgan et al., 2000) (Figure 1A), which was evidenced by a significant main effect of group [*F*_(2, 62)_ = 41.63, *p* < 0.001] and a time × group interaction [*F*_(18, 110)_ = 29.04, *p* < 0.001). Increased levels were observed from 30 min post-administration onwards in both hydrocortisone administration conditions, and the levels remained elevated for at least 90 min. As intended, treatment resulted in elevated cortisol levels during fMRI scanning in the rapid hydrocortisone condition, whereas the levels in the slow condition had already returned to baseline.

Post-experiment debriefing showed that participants were unable to identify the substance received. As expected, hydrocortisone administration did not affect mood as assessed three times during the experiment using the POMS questionnaire (Reddon et al., 1985; Wald and Mellenbergh, 1990; de Groot, 1992) (Table 1). Although significant reductions in levels of depression scores [Friedman's ANOVA; χ^2^_(2)_ = 9.16, *p* = 0.01], anger scores [χ^2^_(2)_ = 7.93, *p* = 0.02], vigor scores [χ^2^_(2)_ = 73.17, *p* < 0.001], and tension scores [χ^2^_(2)_ = 22.41, *p* < 0.001] were observed over the course of the experiment, and levels of fatigue [χ^2^_(2)_ = 48.41, *p* < 0.001] increased, none of these factors were affected by drug administration. Groups did not differ on any aspect of mood state at baseline, nor at any other time point during the experiment (all *p* > 0.1). Changes in mood over time were also not affected by drug administration (all *p* > 0.05). Hence, differences in brain activity found between drug conditions cannot readily be explained by any psychological effects of drug administration.

**Table 1:** **Behavioral performance on the emotional interference task**.

	**Placebo**	**Rapid CORT**	**Slow CORT**
Reaction times neutral, in ms	674(17)	702(23)	650(20)
Reaction times aversive, in ms	687(17)	709(23)	664(20)
Emotional interference on reaction times, in Δms	12(7)	7(7)	14(6)
Correct responses neutral, in %	95.03(1.03)	95.29(0.64)	96.63(0.64)
Correct responses aversive, in %	95.24(0.90)	93.96(0.77)	96.80(0.63)
Emotional interference on correct responses, in Δ%	0.21(0.44)	−1.33(0.50)^*^	0.17(0.77)
–	–	–	–

Mean values (SEM). All groups were similarly affected in their reaction times by emotional interference, displaying slower responses to aversive compared to neutral words. However, the rapid corticosteroid (CORT) group specifically was impaired in its accuracy of responding due to emotional interference. The rapid CORT group made fewer correct responses to the aversive compared to the neutral words than placebo (

* p < 0.05), and this comparison reached a trend for the difference with corticosteroids' slow effects.

### Emotional interference task

Overall task performance, assessing sustained attention by combining results on the neutral and aversive trials, was not significantly affected by hydrocortisone intake. No effects of group were found on reaction times [*F*_(2, 62)_ = 1.49, *p* = 0.233]. Analysis of the error rates, however, seemed to indicate better performance due to the slow effects of corticosteroids. The slow corticosteroid group seemed to make fewer errors than the other groups, but significance just reached trend level [main effect of group: *F*_(2, 62)_ = 2.33, *p* = 0.106, slow CORT vs. placebo: *F*_(1, 41)_ = 2.26, *p* = 0.141, slow CORT vs. rapid CORT: *F*_(1, 42)_ = 6.27, *p* = 0.016]. Processes of sustained attention might thus benefit from the slow effects of corticosteroids.

Next, we tested for the effects of emotion on task performance. As expected, emotion interfered with selective attention. Participants responded significantly slower to aversive words compared to neutral ones [Main effect of emotion (emotional interference): *F*_(1, 62)_ = 9.42, *p* = 0.003]. Emotion did however not significantly affect error rates [*F*_(1, 62)_ < 1] (Table 1).

Hydrocortisone intake had no significant influence on the emotion effect on reaction times [Emotion × group interaction: *F*_(2, 62)_ < 1], but did show a trend for the influence of corticosteroids on the emotion effect on correct response rate [*F*_(2, 62)_ = 2.20, *p* = 0.12]. This trend appeared to be caused by the rapid corticosteroid (CORT) group, which was significantly affected [*T*_(21)_ = −2.65, *p* = 0.015] in its accuracy of responding by emotional interference, whereas both other groups were not (both *p*'s> 0.6). The rapid effects of corticosteroids induced fewer correct responses for the aversive relative to the neutral words than placebo [Emotion group interaction (rapid CORT vs. placebo): *F*_(1, 41)_ = 5.23, *p* = 0.03], and this comparison reached a trend for the difference with corticosteroids' slow effects [Emotion × group interaction (rapid CORT vs. slow CORT): *F*_(1, 42)_ = 2.65, *p* = 0.11)] No such differences were observed between the slow effects of corticosteroids and placebo [Emotion × group interaction (slow CORT vs. placebo): *F*_(1, 41)_ < 1]. Thus, the rapid effects of corticosteroids appeared to increase the susceptibility to emotional interference.

### Brain activation data

We first identified brain regions involved in task execution in comparison to rest (fixation). As expected, task execution recruited a large cluster of brain regions involved in visual processing, including the bilateral middle and inferior occipital lobe, calcarine, cuneus, cerebellum, lingual gyrus, and fusiform gyrus (Table 2). Moreover, brain regions involved in motor and executive function were activated, including the angular, parietal and precentral cortex, and the superior and middle frontal gyrus. Regions deactivated by task execution included regions of the default mode network; the medial PFC (superior, middle, and orbitofrontal cortex), the temporal lobe (covering the hippocampus and amygdala), cingulate gyrus (posterior, middle, and anterior), precuneus and cuneus, and regions within the cerebellum (Table 2).

**Table 2:** **Peak voxels and corresponding *T* values of significantly activated clusters in main effects of task, emotion, and drug**.

	**MNI-coordinates**			**Peak *T*-value**
	***x***	***y***	***z***	
**POSITIVE EFFECT OF TASK**				
Extended cluster covering visual processing areas: inferior, middle, and superior occipital gyrus, calcarine, lingual gyrus, fusiform gyrus, cerebellum	16	−92	−4	8.90^***^
	−16	−92	−8	23.28^***^
Supplemental motor area	−4	8	50	20.12^***^
Middle cingulate cortex				
Precentral cortex, R	32	−56	52	14.15^***^
Superior frontal cortex, R				
Inferior parietal cortex, R				
Angular cortex, R				
Inferior parietal cortex, L	−30	−52	48	19.06^***^
Angular cortex, L				
Precentral cortex, L	−28	−4	54	15.50^***^
Superior frontal cortex, L				
Middle frontal cortex, R	48	38	30	6.95^***^
Middle frontal cortex, L	−34	52	30	5.10^*^
Inferior frontal cortex, L	−40	28	24	5.93^***^
Insula, R	34	24	2	4.91^*^
Insula, L	−32	20	6	7.21^***^
Thalamus, R	12	−16	10	8.37^***^
Thalamus, L	−10	−18	10	10.65^***^
Putamen, L				
Putamen, R	26	4	−6	7.48^***^
Brain stem	−6	−28	−4	5.39^**^
Cerebellum, L	−20	−62	−50	5.66^**^
**NEGATIVE EFFECT OF TASK**				
Activation cluster covering the bilateral angular cortex, middle occipital cortex, cuneus, precuneus, posterior and middle cingulate cortex, middle temporal gyrus, lingual gyrus, parahippocampus gyrus, hippocampus, amygdala	−44	−76	32	17.86^***^
	46	−76	28	
Activation cluster covering the middle frontal cortex, superior frontal cortex, superior medial cortex, anterior cingulate cortex, rectus and middle orbitofrontal cortex	28	26	40	14.05^***^
	−24	30	44	
Inferior frontal cortex, L	−46	42	6	5.02^*^
	−58	32	2	4.96^*^
Middle orbitofrontal cortex, L	−48	50	0	4.91^*^
Insula, R	36	6	12	5.91^***^
Lingual gyrus, L	−14	−60	−4	5.57^**^
Cerebellum, R (Crus2)	44	−66	−40	6.84^***^
Cerebellum, L (Crus2)	−42	−70	−40	5.10^*^
Cerebellum, R (9)	6	−50	−42	5.97^***^
**POSITIVE EFFECT OF EMOTION**				
Inferior frontal cortex and inferior orbitofrontal cortex, L	−44	32	0	5.95^***^
Superior temporal pole, L	−58	6	−10	5.57^**^
Middle temporal pole, L	−52	14	−24	5.21^*^
**MAIN EFFECT OF DRUG**				
*Placebo > slow CORT*				
Cuneus, L	−18	−72	36	5.51^**^

The peak x, y, z coordinates are given in MNI152 standard space coordinates. L and R denote left and right. Main effects of task are all thersholded at p < 0.05 FWE corrected at the voxel-level.

* , p < 0.05;

** , p < 0.01;

*** , p < 0.001.

Subsequently, we tested for the effect of emotion during task execution. Regions that were more active during the processing of aversive compared to neutral words were mainly language-related areas in the left inferior frontal cortex (BA45), left inferior orbitofrontal cortex, superior temporal pole, and the middle temporal lobe (BA38). No regions were more active during the processing of neutral compared to aversive words (Table 2).

Next, we examined how corticosteroids affected sustained attentional processing. Looking into the main effect of drug (contrasting all three drug conditions) revealed a main effect in the cuneus [(−18, −72, 36), *F*_(2, 124)_ = 16.36, *p* = 0.022], which was driven by reduced activity due to the *slow* effects of corticosteroids [placebo > slow CORT: (−18, −72, 36), *T*_(124)_ = 5.51, *p* = 0.004]. Under basal (i.e., placebo) conditions this part of the cuneus was activated during task-execution, suggesting its involvement in visual processing (Hahn et al., 2006), but the slow effects of corticosteroids reduced its activation. In contrast, we did not find any main effect on brain processing in the rapid corticosteroid condition.

**Figure 2:**
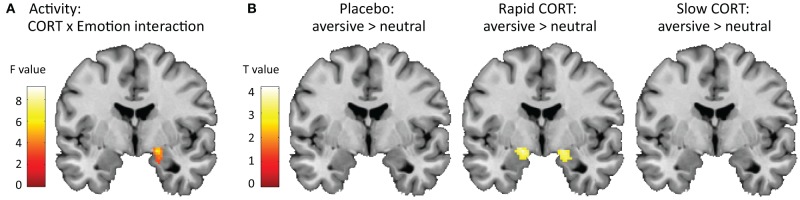
**Effects of corticosteroids on amygdala activity. (A)** Hydrocortisone administration induced trend of a corticosteroid *x* emotion interaction in the amygdala (*y* = −4). For visualization purposes the statistical parametric map is thresholded at *p* < 0.05 uncorrected with a minimal cluster-size of 250 voxels. **(B)** This interaction appeared to be driven by a significant effect of emotion in the amygdala due to the rapid effects of corticosteroids, suggesting insufficient suppression of emotional interference in this group. The amygdala in the placebo and slow corticosteroid group did not distinguish between the processing of aversive vs. neutral words. For visualization purposes the statistical parametric maps are thresholded at *p* < 0.005 uncorrected with a minimal cluster-size of 25 voxels.

To test how corticosteroids influenced selective attention, or emotional interference, we next checked for a drug × emotion interaction in the brain. Indeed, we found a trend toward such interaction in the amygdala specifically [(20, −4, −16), *F*_(2, 124)_ = 5.02, *p*_SVC_ = 0.077] (Figure 2A). This interaction appeared to be driven by an increased effect of emotional interference due to the *rapid* effects of corticosteroids [Emo(rapid CORT) > Emo(placebo): (20, −4,−16), *T*_(124)_ = 3.15, *p*_SVC_ = 0.037; Emo(rapid CORT) > Emo(slow CORT): (22, −4, −18), *T*_(124)_ = 2.49, *p*_SVC_ = 0.071]. Whereas amygdala responsivity with placebo or under the influence of slow effects of corticosteroids did not distinguish between neutral and aversive words, suggesting sufficient suppression of emotional interference, the rapid effects of corticosteroids-induced significantly higher amygdala responses while color-naming aversive compared to neutral words [(22, −2, −16), *T*_(124)_ = 3.96, *p*_SVC_ = 0.036] (Figure 2B). Thus, the increase in emotional interference observed in behavioral performance due to the rapid corticosteroid effects, was reflected in the brain as an enhanced emotion effect in the amygdala, indicating failed suppression of emotional processing.

### Brain connectivity data

Next, we assessed whether the corticosteroid-induced alterations in amygdala responses were related to any changes in functional connectivity of this region to the rest of the brain. First, brain regions were identified that were functionally coupled, i.e., displaying significantly correlated time courses of activity, to the amygdala across all drug conditions. Activity in the amygdala was positively associated to activity in a large cluster covering the bilateral amygdala itself, thalamus, pallidum, putamen, hippocampus, parahippocampal gyrus, fusiform, middle and superior temporal lobe, insula, and inferior, middle, and superior orbitofrontal cortex. Other regions positively associated with amygdala activity included the brain stem (including the LC), regions within the anterior and middle ACC, superior frontal cortex, and regions within the cerebellum (Table 3). Conversely, amygdala activity was negatively associated with activity in frontal regions such as the medial superior frontal gyrus, superior, middle, and inferior frontal gyrus, and regions within the anterior and middle ACC, and with the insula, brain stem, and cerebellum. Overall, these patterns of functional connectivity are in line with previous studies (Roy et al., 2009; van Marle et al., 2010; Henckens et al., 2011b) and support models of emotion processing that suggest reciprocal ventral and dorsal systems (Phillips et al., 2003). However, one remarkable difference is the negative coupling of the amygdala to the insula observed in this study. This might suggest that the insula, during task execution, is functioning as part of an executive network (Binder et al., 2004; Nee et al., 2007) instead of the salience network (Seeley et al., 2007).

**Table 3:** **Peak voxels and corresponding *T* values of significantly activated clusters that show functional coupling with the bilateral amygdala**.

	**MNI-coordinates**			**Peak *T*-value**
	***x***	***y***	***z***	
**POSITIVE OVERALL AMYGDALA COUPLING**				
Extended cluster covering the bilateral amygdala, brainstem (LC), thalamus, pallidum, putamen, hippocampus, parahippocampal gyrus, fusiform gyrus, middle and superior temporal lobe, inferior, middle and superior orbitofrontal cortex, anterior cingulate cortex and cerebellum	22	−2	−16	43.38^***^
Superior frontal cortex, R	20	70	8	5.45^*^
Middle cingulate cortex	0	0	46	5.50^*^
Caudate, L	−8	16	22	6.83^***^
Thalamus, R	16	−20	16	5.66^*^
Midbrain	14	−28	−26	7.99^***^
Cerebellum Crus2, L	−36	−80	−36	6.45^***^
Crus2, L				
**NEGATIVE OVERALL AMYGDALA COUPLING**				
Anterior and middle cingulate cortex, superior medial cortex, R	2	28	10	9.85^***^
Middle cingulate cortex, R	22	−16	32	9.08^***^
Middle cingulate cortex, L	−24	−4	36	9.80^***^
Inferior frontal gyrus, R	64	18	18	5.81^**^
Inferior frontal gyrus, L	−52	28	22	9.19^***^
Inferior frontal gyrus, L	−42	14	32	5.40^*^
Inferior and middle frontal gyrus, L	46	40	26	6.57^***^
Middle frontal gyrus, L	−26	48	30	5.59^*^
Middle and superior frontal gyrus, L	−20	54	30	5.56^*^
Superior frontal gyrus, R	20	54	32	7.37^***^
Superior frontal gyrus, R	20	18	56	5.81^**^
Insula, R	−44	−2	6	8.70^***^
Insula, L	44	−4	4	8.86^***^
Thalamus, R	0	−18	6	9.42^***^
Middle temporal gyrus, R	58	−42	4	7.60^***^
Parahippocampal gyrus, R	16	−28	−16	8.20^***^
Inferior occipital and lingual gyrus, R	36	−86	−6	7.02^***^
Inferior occipital and lingual gyrus, middle temporal gyrus, L	−26	−90	−4	7.16^***^
Inferior parietal cortex, L	−50	−50	36	5.53^*^
Cerebellum, L	−18	−30	−18	5.85^**^
Cerebellum and brain stem, L	−16	−44	−28	13.39^***^
Brain stem	0	−8	−16	5.72^*^

The peak x, y, z coordinates are given in MNI152 standard space coordinates. L and R denote left and right. Overall amygdala coupling is thresholded at p < 0.05 FWE corrected at the voxel-level,

* , p < 0.05;

** , p < 0.01;

*** , p < 0.001 whole-brain corrected.

**Figure 3:**
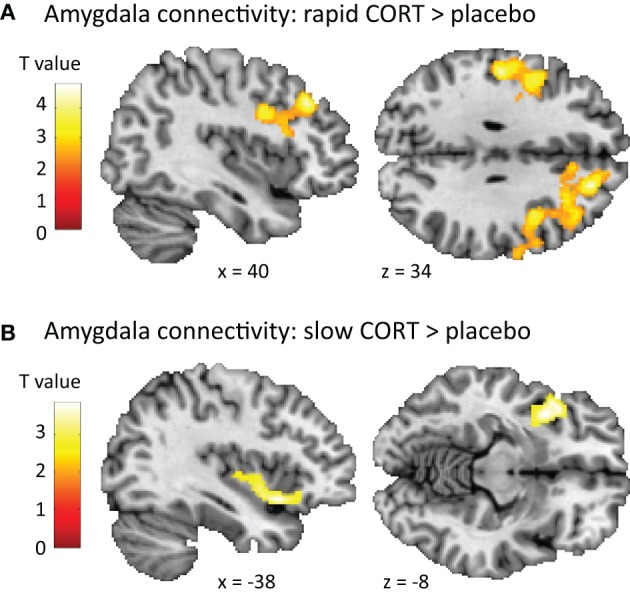
**Effects of corticosteroids on amygdala connectivity. (A)** The rapid effects of corticosteroids increased the functional connectivity of the amygdala to regions involved in task execution (middle frontal gyrus, precentral gyrus, and postcentral gyrus), potentiating its influence on task execution. **(B)** The slow effects of corticosteroids disrupted the negative connectivity between the amygdala and insula, attenuating the effects the amygdala can exert on task execution. For visualization purposes the statistical parametric maps are thresholded at *p* < 0.01 uncorrected with a minimal cluster-size of 250 voxels.

Second, when contrasting connectivity patterns between drug conditions, the *rapid* effects of corticosteroids influenced amygdala connectivity to regions involved in task execution including the middle frontal and precentral gyrus [(42, 26, 40), *T*_(62)_ = 4.79, *p* < 0.001], and the postcentral gyrus [(−52, −20, 34), *T*_(62)_ = 4.39, *p* = 0.005] (Figure 3A). Whereas these structures displayed *negative* connectivity with the amygdala under basal (i.e., placebo) conditions, the rapid corticosteroid effects induced *positive* connectivity between the amygdala and this executive network. In addition, the slow effects of corticosteroids altered amygdala connectivity to the left insula [(−38, 10, −8), *T*_(62)_ = 3.85, *p* = 0.001]. The negative amygdala-insula coupling observed under placebo conditions was weakened by the slow effects of corticosteroids (Figure 3B).

## Discussion

In this study we investigated the time-dependent effects of corticosteroids on selective attention and emotional interference. The results suggest that the rapid effects of corticosteroids specifically increased emotional interference in terms of error rate, which was associated with reduced amygdala inhibition to aversive words. Moreover, they induced enhanced amygdala connectivity with frontoparietal brain regions, possibly reflecting increased influence of the amygdala on an executive control network. In contrast, the slow corticosteroid effects seemed to modulate the neural correlates of sustained attention by decreasing cuneus' activity, potentially indicating reduced stimulus-driven (bottom-up) attentional processing. Furthermore, they altered the coupling of the amygdala to the insula, which might affect emotional interference. Thus, corticosteroids seemed to modulate different aspects of attentive processing in a time-specific manner.

Previous animal work has indicated that corticosteroids, next to their well-established slow genomic effects, also exert rapid non-genomic effects on brain function (Joels et al., 2006). In the amygdala, the hormones have been shown to rapidly affect neuronal plasticity by binding to MR, leading to an increase in glutamate release (Karst et al., 2010). At the same time, the binding of primarily intracellular GRs initiates a corticosteroid-induced genomic cascade that modulates the expression of over 200 genes (Datson et al., 2001). Here, we aimed to dissociate these two effects experimentally by administering 10 mg of hydrocortisone at either 60 or 270 min prior to the emotional distraction task. The timing of the rapid corticosteroid condition was based (A) on a previous study in our lab revealing an elevation in human salivary cortisol levels from 30 min after hydrocortisone intake onwards (Henckens et al., 2011a), (B) previous rodent studies revealing a ~20 min delay between elevations in corticosteroid levels in plasma and brain (Droste et al., 2008), and (C) rapid effects of corticosteroids administered directly to amygdala slices in rodents from ~10 min post administration onwards (Karst et al., 2005). The genomic effects of corticosteroids on the other hand generally do not start earlier than at least 3 h after exposure to high corticosteroid levels *in vivo* (Joels et al., 2003; Morsink et al., 2006) and these effects last for hours (Joels and de Kloet, 1992; Joels et al., 2003). Thus, administration of hydrocortisone at 60 min prior to scanning probably caused sufficiently high levels of the hormone in the brain to evoke rapid non-genomic effects whereas this delay was too short to allow development of gene-mediated events. Conversely, when hydrocortisone was applied at 270 min prior to testing, hormone levels were back to baseline levels again during the behavioral task, making non-genomic actions not likely to happen, yet allowed enough time for the gene-mediated actions to occur. For these reasons, the rapid corticosteroid effects observed here most likely reflect corticosteroid's non-genomic effects, whereas the slow corticosteroid effect most likely involve a gene-mediated mechanism, although obviously this cannot be proven in the human brain.

Here we showed that the rapid corticosteroid effects increase emotional interference. Participants had difficulty ignoring emotional input; they made more mistakes for the aversive words and failed to down-regulate their amygdala response to this input. These findings are in line with the hypothesis that the rapid effects of corticosteroids act in concert with catecholamines in response to stress to optimize rapid adaptive behavior (Roozendaal et al., 2006; Diamond et al., 2007). Previous studies have already shown that during acute stress, the brain switches into a hypervigilant stimulus-driven reflex-like mode of processing, characterized by heightened overall attention, but also by increased susceptibility to (emotional) distraction (Skosnik et al., 2000; Braunstein-Bercovitz et al., 2001; Henderson et al., 2012) and impaired flexibility (Plessow et al., 2012). Performance on relatively easy (e.g., perceptual) tasks seems to benefit by this state of increased arousal, but performance on more difficult tasks requiring executive control seems to deteriorate (Jasinska et al., 2012; Lee et al., 2012). Recent neuroimaging studies have indicated that this hypervigilant brain state is associated with enhanced sensory processing (Henckens et al., 2009), increased amygdala responsivity to emotional input (van Marle et al., 2009) and tightened amygdala connectivity to the salience network (van Marle et al., 2010). Moreover, PFC function gets deteriorated (Qin et al., 2009). This state-change of brain processing has previously been attributed to the actions of catecholamines on brain function (Arnsten and Li, 2005; Hermans et al., 2011; Qin et al., 2012). Our findings of increased emotional interference indicate that, next to the effects of catecholamines, the rapid effects of corticosteroids also contribute to this state of hypervigilance.

Earlier animal work already indicated that corticosteroids' rapid non-genomic effects, mediated by membrane-bound steroid receptors, boost amygdala activity (Kavushansky and Richter-Levin, 2006; Karst et al., 2010), while impairing PFC function (Barsegyan et al., 2010). Next to that, evidence for corticosteroid-modulation of noradrenergic function is abundant, both in animal (Roozendaal et al., 2006; McReynolds et al., 2010; Zhou et al., 2012) and human research (van Stegeren et al., 2007, 2010). Recent drug administration studies in humans for example showed that corticosteroid administration in combination with the administration of reboxetine (a noradrenaline-reuptake inhibitor) induced a negative response bias in the amygdala (Kukolja et al., 2008), and boosted emotion-induced retrograde amnesia (Hurlemann et al., 2007), in line with our findings of increased distraction by aversive input and increased susceptibility to the effects of emotion, respectively.

However, administration of hydrocortisone on brain function has produced quite conflicting results. One potential confounding factor is the type of brain function investigated in these studies. A widely accepted phenomenon from memory research for example is that corticosteroids influence processes of memory encoding and consolidation in an opposite manner than memory retrieval (Roozendaal, 2002), although both processes heavily depend on hippocampal function. Nevertheless, corticosteroids boost memory encoding and associated hippocampal activation (van Stegeren et al., 2010), whereas they impair hippocampal activation during memory retrieval (de Quervain et al., 2003). Similarly, differential effects of corticosteroids have been observed depending on the function studied of the PFC (Henckens et al., 2011a,c) and amygdala (Henckens et al., 2010; Lovallo et al., 2010; Tabbert et al., 2010; van Stegeren et al., 2010). Another crucial factor possibly explaining the discrepancies between studies is the dose in which hydrocortisone was administered (Lupien et al., 2007). Previous research has indicated that corticosteroids influence memory processing in an inverted U-shaped relationship (Lupien et al., 1997), with both low and high doses impairing memory consolidation, while moderate levels improve consolidation (Roozendaal, 2000). Also the effects of corticosteroids on working memory (Lupien et al., 1999) and startle response (Buchanan et al., 2001) have been shown to be dose-dependent. This non-monotonic relationship between corticosteroids and their effects on cognitive function is hypothesized to be related to the differential activation of the MRs and GRs, which show distinct affinity for the hormone (de Kloet, 2003). The doses used in previous research range from 10–100 mg of hydrocortisone, and obviously produce different results. A recent review on the immediate effects of corticosteroids on selective attention concluded that corticosteroids actually facilitate stress-coping via the inhibition of autonomic processing of goal-irrelevant threatening information, when administered in a dose of >35 mg (Putman and Roelofs, 2011). The authors admit that lower doses might lead to different results. Here, we used a dose of 10 mg of hydrocortisone to mimic cortisol elevations in response to a moderate-to-severe stressor, and show that the rapid effects of corticosteroids increase emotional interference during executive function.

Moreover, the rapid effects of corticosteroids also affected amygdala connectivity. Connectivity to the middle frontal gyrus and precentral and postcentral gyrus was increased 60 min after hydrocortisone administration. Being part of an executive and motor network, these regions were recruited during task execution (Table 2). Whereas the pre- and postcentral gyrus are involved in more basic motor functions, the middle frontal gyrus is known for its role in response selection and suppression of automatic response tendencies (Forstmann et al., 2008), as well as in resolving interference (Nee et al., 2007). Under basal (i.e., placebo) conditions, all of these regions were negatively coupled to the amygdala, underlining their opposing roles in task execution. In contrast, the rapid effects of corticosteroids led to positive coupling between the amygdala and the executive network. Although one cannot infer any directionality from such correlative evidence, this might be suggestive for increased influence of the amygdala on brain regions crucially involved in task execution. This interpretation of the data would fit with the increase in emotional interference, but future research is needed to test this assumption.

Besides these rapid effects of corticosteroids on emotional interference we showed that the slow effects of corticosteroids modulated the neural correlates of sustained attention by reducing activity of the cuneus. This brain region is involved in basic visual processing, and has been shown to be engaged by stimulus-driven, bottom-up attentional processing (Hahn et al., 2006). Previous research has indicated that acute stress boosts visual processing (Henckens et al., 2009; van Marle et al., 2009), and more specifically, the rapid effects of corticosteroids have been shown to increase cuneus' regional cerebral activity during rest (Ganguli et al., 2002; Strelzyk et al., 2012). These data suggest that stress, or the rapid effects of corticosteroids, boost early visual processing and thereby shift the brain into a rather automated visually guided response-mode, which serves the fight-or-flight response. The slow effects of corticosteroids might in turn counteract these effects by reducing cuneus' activity, and shifting the brain back from a stimulus-driven response mode to a more controlled mode. This rationale fits with the general idea about the restorative role the slow corticosteroid effects serve in the aftermath of stress in order to return to homeostasis (de Kloet et al., 2005). The slow effects of corticosteroids have been shown to divert energy supply to challenged tissues and control the excitability of neuronal networks (de Kloet et al., 2008). Evidence from recent human neuroimaging studies also supports this hypothesis by showing that corticosteroids' slow effects are the exact opposite of those of acute stress. Whereas acute stress impairs PFC function (Qin et al., 2009) and boosts amygdala activity (van Marle et al., 2009), the slow effects of corticosteroids' enhanced PFC function (Henckens et al., 2011c) and suppressed amygdala responsivity to faces (Henckens et al., 2010). Here, we showed that the slow effects of corticosteroids reduced cuneus' activity, which might be another means to restore proper brain function in the aftermath of stress.

The slow effects of corticosteroids also reduced the negative connectivity between the amygdala and left anterior insula, seen under placebo conditions. The amygdala and anterior insula share widespread reciprocal connections (Mufson et al., 1981), and are known for their role in mediating autonomic arousal as part of the so-called salience network (Seeley et al., 2007). Connectivity in this network is known to be increased by acute stress (van Marle et al., 2010; Hermans et al., 2011) and serve the fight-or-flight response by promoting the information exchange between regions involved in autonomic-neuroendocrine control and vigilant attentional reorienting. However, next to the typical link to cortical control of autonomic function, the insula is consistently reported to be activated during experiments in which task conditions are challenging, and decisions have to be made (Binder et al., 2004). Therefore, it was recently suggested (Eckert et al., 2009) that the anterior insula engages brain regions selectively responsive to task demands and attention systems critical for coordinating task performance. In line with this hypothesis, a recent meta-analysis on neuroimaging studies into the resolution of interference pointed toward the involvement of the anterior insula in resolving interference (Nee et al., 2007). Although one cannot infer directionality from the correlative analysis performed, one could speculate that the negative connectivity between the amygdala and insula observed in our experiment reflects the interference of the amygdala with proper task performance. By reducing this connectivity, the slow effects of corticosteroids might attenuate the effect the amygdala can exert on task execution. Therefore, also the reduced amygdala-insula connectivity could entail a mechanism by which the slow effects of corticosteroids restore brain function in the aftermath of stress. However, this interpretation should be tested in future research.

Some limitations to the study should also be mentioned. First of all, this study involved a pharmacological manipulation to model the effects of corticosteroids, which does obviously not capture all aspects of the complex stress response. Real-life cortisol release in response to stress is accompanied by the release of many other neuromodulators, such as NE, corticotrophin-releasing hormone, dopamine, and serotonin (Joels and Baram, 2009), with which corticosteroids could potentially interact. Because we did not induce stress, the generalization from our results to stressful situations remains speculative. Nevertheless, mere administration of hydrocortisone reveals a cleaner mechanistic account for the corticosteroid effect, which was the aim of this study.

Secondly, we investigated men only, thus the obtained results cannot be readily generalized to women. Hydrocortisone administration has been shown to induce distinct effects in men and women, both in behavior (Andreano and Cahill, 2006; Bohnke et al., 2010) and brain activation (Stark et al., 2006; Merz et al., 2010). Although important, sex-differences were beyond the scope of this initial study, which is why we opted to recruit male subjects only, allowing easier comparison with an earlier study in stressed individuals (Henckens et al., 2009).

Furthermore, the increase in emotional interference by the rapid effects of corticosteroids was only observed in terms of error rate (i.e., lower correct response rate) and not in terms of reaction times (i.e., slower responding). On the other hand, the overall effect of emotion was only observed for reaction times. One could suggest that these findings reflect a shift in response strategy induced by the rapid effects of corticosteroids rather than an increase in emotional interference (Chen and Johnson, 1991). This would mean that the rapid CORT group shifted from an accuracy-driven strategy, affecting reaction times while optimizing accuracy, toward a speed-driven strategy, affecting error rates but optimizing speed. However, besides the observed differences (i.e., increase in emotional interference) in error rate one would then also expect differences (i.e., reduced emotional interference effect) in reaction times. This does not seem to be the case. No differences between groups in overall reaction times [*F*_(2, 62)_ = 1.49, *p* = 0.23], nor emotional interference in reaction times [*F*_(2, 62)_ < 1] were observed, indicating that the rapid CORT group is not different from the other groups in terms of reaction times. In terms of error rate, the rapid CORT group was significantly affected, indicating increased emotional interference in this group. Moreover, if it would be the case that the rapid CORT group shifted away from an accuracy-driven toward a speed-driven strategy, one would expect faster responding in this group, which is also not observed. All in all, it is difficult to speculate about the reason why we did find interference effects in one measure and not the other. However, small behavioral effects are not unprecedented in previous studies (Haas et al., 2006; Mincic, 2010). Importantly, behavioral emotional interference effects are most consistently observed in psychopathological groups in response to words that are specific to their disorder (Dalgleish, 1995; Williams et al., 1996), and in normal subjects when the words are related to current concerns endorsed by them (Gilboa-Schechtman et al., 2000), reflecting their attentional bias. Overall, in normal subjects, behavioral interference by emotional distracters is either not detected at all (Williams et al., 1996), is depending on specifc personality traits such as trait anxiety (Richards et al., 1992; Krug and Carter, 2010) or extraversion (Haas et al., 2006) or habituates rapidly (McKenna, 1986; Compton, 2003). We used rather general aversive words, non-specific to the participants, which might explain why we only find overall effects in terms of reaction times and not error rate. Nevertheless, emotional interference can express itself in both reaction times and number of errors (Swick and Jovanovic, 2002; Wagner et al., 2006; Weiss et al., 2007; Kertzman et al., 2010; Crocker et al., 2012).

Furthermore, the slow effects of corticosteroids manifested themselves only as altered brain activity, without translating to behavioral differences. The most likely explanation for the absence of a (clear) behavioral effect might be a lack of power of our neuroimaging study. Compared to behavioral studies, which tend to test large groups of subjects, our sample size is relatively small. Brain activity is supposed to be a more sensitive measure than behavioral output, which is the consequence of many parallel neural operations. Therefore, regional differences in brain activity are more easily detected with smaller samples, but these samples offer little power to observe behavioral effects. However, a trend toward a better overall performance due to the slow effects of corticosteroids was observed in the behavioral data, since the slow corticosteroid group tended to make fewer errors than the other groups. These data therefore seem to support the enhanced sustained attention due to the slow effects of corticosteroids, but future studies using larger sample sizes are needed to confirm these effects.

Lastly, we interpreted the effects of emotional interference as a measure of selective attention, because this condition requires the attentional selection of relevant features while ignoring competing information. However, these findings cannot be readily generalized to other selective attention tasks. The emotional component might be critical in interfering with attentional processing, as corticosteroids have been shown to exert more prominent effects on the processing of emotional compared to neutral information (Roozendaal et al., 2006). Therefore, future studies are necessary to determine whether the rapid effects of corticosteroids can be regarded as generally or emotion-specifically interfering with the neural processing of selective attention.

In conclusion, these results suggest that the rapid effects of corticosteroids increase emotional interference and selective attention. Although increased susceptibility to interference, and thus impaired selective attention, is often seen as a maladaptive response of attenuating higher-cognitive function, it is first and foremost a highly adaptive response in threatening situations. Wide-spread, unfocussed attention might contribute to the detection of potential threats in the environment (Aston-Jones and Cohen, 2005), enhancing an organism's chances of survival. Moreover, it might have beneficial effects on memory processing (Henckens et al., 2009), since additional environmental cues can also be encoded during a salient event. Normalization some time after the stressful event is equally important. When not properly regulated, the increased processing of irrelevant emotional input due to combined corticosteroid and noradrenergic actions as well as the lack of normalization can be detrimental. Patients with stress-related disorders such as depression and PTSD are known to be compromised in their capability to suppress emotional irrelevant information (Paunovi et al., 2002; Mitterschiffthaler et al., 2008), which is thought to reflect their attentional bias toward negative emotional information (Williams et al., 1996). Notably, these illnesses are characterized by aberrant corticosteroid signaling (Yehuda et al., 2001). Our results provide thus a mechanistic account for these problems with attention and emotional interference, by showing that the rapid effects of corticosteroids interfere with amygdala function, and the slow effects modulate the neural correlates of sustained attention.

### Conflict of interest statement

The authors declare that the research was conducted in the absence of any commercial or financial relationships that could be construed as a potential conflict of interest.
